# Incidental Tc-99m Methylene Diphosphonate Uptake in an Active Thyroid Nodule

**DOI:** 10.4274/mirt.37167

**Published:** 2017-10-02

**Authors:** Derya Çayır, Mine Araz, Şafak Akın, Melia Karaköse, Erman Çakal

**Affiliations:** 1 University of Health Sciences, Dışkapı Yıldırım Beyazıt Training and Research Hospital, Clinic of Nuclear Medicine, Ankara, Turkey; 2 University of Health Sciences, Dışkapı Yıldırım Beyazıt Training and Research Hospital, Clinic of Endocrinology and Metabolism, Ankara, Turkey

**Keywords:** Thyroid nodule, Tc-99m medronate, Radionuclide imaging

## Abstract

Tc-99m-methylene diphosphonate (MDP) whole body scintigraphy is the method of choice for detection of metastatic bone diseases. It is primarily used to help diagnose various bone-related conditions such as primary or metastatic cancer of the bone, location of bone inflammation, and fractures that may not be visible on traditional X-ray images, as well as detection of bone damage due to infections and other conditions. In addition, bone scanning is often used for the follow-up or evaluation of response to treatment in some malignancies like prostate and breast cancers. Pathologies of other systems can also be incidentally detected on whole body bone scan. Herein we present an interesting image of an active thyroid nodule that showed Tc-99m MDP uptake in a prostate cancer patient.

## Abstract (tr)

Tc-99m metilen difosfonat (MDP) tüm vücut kemik sintigrafisi kemik metastazlarının araştırılmasında sıklıkla uygulanan bir tetkiktir. Primer ve metastatik kemik kanseri tanısı, kemik enflamasyonunun lokalizasyonu, geleneksel X-ray görüntülerinde izlenemeyen fraktürlerin tanısı, enfeksiyon ve diğer durumlara bağlı gelişebilen kemik hasarının tanısı gibi kemik ile ilişkili çok sayıda durumun teşhisinde yardımcıdır. Ek olarak, kemik sintigrafisi, prostat ve meme kanseri gibi bazı malignitelerin takibinde veya tedaviye yanıtının değerlendirilmesinde sıklıkla kullanılmaktadır. Tüm vücut kemik sintigrafisinde diğer sistemlere ait patolojiler insidental olarak saptanabilir. Bu olguda prostat karsinomu tanısı olan hastada Tc-99m MDP tutan aktif tiroid nodülüne ait ilginç görünümü sunuyoruz.

## Figures and Tables

**Figure 1:**
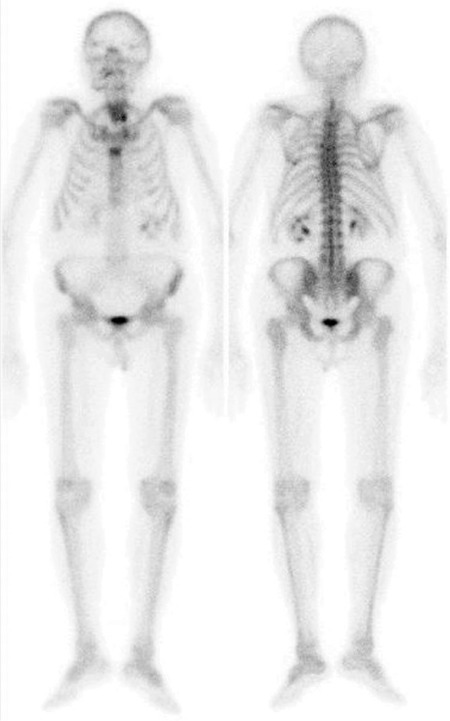
A 84-year-old male patient with prostate adenocarcinoma underwent technetium-99m (Tc-99m) methylene diphosphonate (MDP) whole body bone scintigraphy in order to evaluate bone metastasis. The patient did not have any other known diseases or complaints. The bone scintigraphy showed focal increased activity in the area consistent with the inferior pole of the left lobe of the thyroid gland. The serum thyroid function tests were as follows: TSH 2.45 uIU/mL (N: 0.34-5.60), fT3: 3.52 pg/mL (N: 2.5-3.9), fT4: 0.76 ng/dL (N: 0.58-1.6) and thyroid autoantibodies were negative

**Figure 2:**
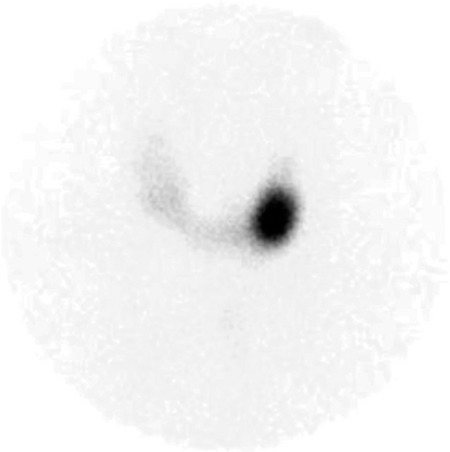
Tc-99m pertechnetate thyroid scintigraphy showed an active nodule in the lower pole of the left lobe, significant suppression was observed in extranodal areas

**Figure 3:**
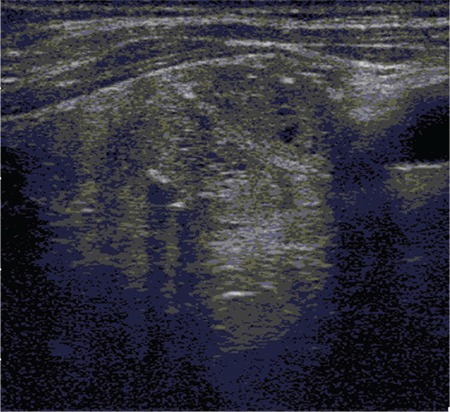
Thyroid ultrasound revealed a 14x14x22 mm nodule with a mixed echo pattern, rough calcification foci and cystic-necrotic areas in the left lobe lower pole. Fine needle aspiration biopsy of this nodule complied with benign follicular nodule. Bone scintigraphy is usually performed to evaluate a wide variety of skeletal abnormalities (1). Tc-99m-labeled diphosphonates have been used for bone scanning as a major diagnostic tool since the beginning of 1970s (2). Tc-99m MDP has rapid blood clearance, excellent in vivo chemical stability, and a high bone-to-soft tissue ratio, thus, it is ideal for bone imaging (3). In the literature, many cases of incidental Tc-99m-MDP uptake by the soft tissue have been reported due to various reasons, both benign (tumoral calcinosis, myositis ossificans) and malignant (sarcomas, adenocarcinomas, metastases) conditions (4,5). Mechanisms leading to increased extraosseous Tc-99m MDP uptake include extracellular fluid expansion, enhanced local vascularity and permeability, and high tissue calcium concentration. The composition of the calcium deposition and the presence of other elements (e.g. iron and magnesium) are important (4). It is known that there may be incidental Tc-99m MDP uptake in the thyroid gland, in calcific thyroid nodules, secondary to biopsy interventions, anaplastic thyroid carcinoma or metastatic thyroid cancer (4,6). In our case, unexpected incidental Tc-99m MDP involvement was presented in a functionally active thyroid nodule. This appearance of Tc-99m MDP uptake in an active thyroid nodule, first demonstrated in this case, is thought to be secondary to the presence of microcalcifications in the nodule
